# Transcriptome Profiling Reveals Effects of Drought Stress on Gene Expression in Diploid Potato Genotype P3-198

**DOI:** 10.3390/ijms20040852

**Published:** 2019-02-15

**Authors:** Xiaohui Yang, Jie Liu, Jianfei Xu, Shaoguang Duan, Qianru Wang, Guangcun Li, Liping Jin

**Affiliations:** 1 Institute of Vegetables and Flowers, Chinese Academy of Agricultural Sciences/Key Laboratory of Biology and Genetic Improvement of Tuber and Root Crop, Ministry of Agriculture and Rural Affairs, Beijing 100081, China; xiaohuiy_0601@163.com (X.Y.); liujie@sinopotato.com (J.L.); xujianfei@caas.cn (J.X.); duanshaoguang@caas.cn (S.D.); wangqianru_caas@163.com (Q.W.); 2 Institute of Vegetables and Flowers, Shandong Academy of Agricultural Sciences/Molecular Biology Key Laboratory of Shandong Facility Vegetable, Jinan 250100, China; 3 National Vegetable Improvement Center Shandong Sub-Center/Huang-Huai-Hai Region Scientific Observation and Experimental Station of Vegetables, Ministry of Agriculture and Rural Affairs, Jinan 250100, China

**Keywords:** potato, diploid, drought, response gene

## Abstract

Potato (*Solanum tuberosum* L.) is one of the three most important food crops worldwide; however, it is strongly affected by drought stress. The precise molecular mechanisms of drought stress response in potato are not very well understood. The diploid potato genotype P3-198 has been verified to be highly resistant to drought stress. Here, a time-course experiment was performed to identify drought resistance response genes in P3-198 under polyethylene glycol (PEG)-induced stress using RNA-sequencing. A total of 1665 differentially expressed genes (DEGs) were specifically identified, and based on gene ontology and Kyoto Encyclopedia of Genes and Genomes (KEGG) analysis, the transcription factor activity, protein kinase activity, and the plant hormone signal transduction process were significantly enriched. Annotation revealed that these DEGs mainly encode transcription factors, protein kinases, and proteins related to redox regulation, carbohydrate metabolism, and osmotic adjustment. In particular, genes encoding abscisic acid (ABA)-dependent signaling molecules were significantly differentially expressed, which revealed the important roles of the ABA-dependent signaling pathway in the early response of P3-198 to drought stress. Quantitative real-time PCR experimental verification confirmed the differential expression of genes in the drought resistance signaling pathway. Our results provide valuable information for understanding potato drought-resistance mechanisms, and also enrich the gene resources available for drought-resistant potato breeding.

## 1. Introduction

Along with the rapid expansion in population and global warming, water scarcity has become a global challenge for agriculture. Drought caused by water shortage seriously affects plant growth and development, and has become one of the main constraints to the production of crops including potato (*Solanum tuberosum*), the third most common crop in the world behind rice and wheat. Potato is a typical temperate climate crop, and it is mainly produced in arid or semi-arid areas with low rainfall [1]. Drought stress not only affects potato growth and development, but also seriously reduces potato tuber quality and yield [2]. Hence, improving drought tolerance and breeding drought-resistant cultivars are very important goals in potato production. Moreover, the elucidation of the molecular mechanisms of drought resistance and identification of drought-resistance genes from resistant materials can provide the theoretical basis and gene resources for breeding drought-resistant varieties.

Plant cells perceive stress stimuli through changes in turgor pressure or membrane receptor activity. These extracellular signals are then converted into intracellular signals through the generation of second messengers that activate transcription factors (TFs) or protein kinases (PKs), leading to the differential expression of specific genes, the accumulation of stress-induced gene products, and finally, to protection against stress [3]. The complex gene expression cascades activated during the response to dehydration stress involve abscisic acid (ABA)-dependent and ABA-independent signaling pathways, which are regulated by the ABA-responsive element-binding protein/ABA-responsive element-binding factors (AREB/ABFs) and dehydration responsive element binding (DREB) factors, respectively [4]. AREB/ABF TFs bind to the ABA-responsive element (ABRE) and have pivotal functions in ABA-dependent gene expression. Similarly, DREB TFs bind to the dehydration-responsive element/C-repeat (DRE/CRT) and play key roles in ABA-independent gene expression in response to osmotic stress.

Research into the mechanisms of drought resistance in potato has lagged behind that in other crops because of its autotetraploid properties. So far, only a few studies have reported the identification of genes responsive to drought stress in potato [5]. Silencing of a nuclear cap-binding protein gene *CBP80* in the cultivar, Desiree increased tolerance to water deficit, and the *MYB33* and/or *MYB101* genes, which act downstream of *CBP80*, are involved in ABA-mediated responses to drought [6]. Some microRNAs, including miR171, miR159, miR166, and miR482, have been characterized for their involvement in drought stress response [7,8]. Comparison of two Andean potato varieties, Sullu and Negra Ojoosa, revealed that more ABA responsive TFs, including WRKY1, were induced during drought stress in the cultivar with less drought resistance, Negra Ojoosa [9]. Later, with the release of the potato reference genome sequence, it became possible to identify drought-resistance genes genome-wide [10]. A number of local drought-resistant potato cultivars, Longyan 3, Ningyan 4, Gwiazda, and Tajfun, which are grown in Gansu and Ningxia in China and in Poland were used for comparative gene expression studies using RNA-seq. Thousands of drought response genes were differently expressed; these genes encode TFs and proteins involved in proline metabolism, antioxidant defense systems, flavonoid biosynthesis, and plant hormone signal transduction [11,12]. In the latest study, 22 potato drought-responsive genes were identified, including mitogen-activated protein kinase kinase kinase 15 (MAPKKK15), a carbohydrate transporter, and nonspecific lipid transfer protein type 2 (nsLPT) [13].

However, the findings of the above studies are still insufficient for revealing the molecular mechanism of potato drought resistance. The lack of studies is likely due to the complexity of the genetic background of potato cultivars, which are autotetraploids (2n = 4x = 48) and highly heterozygous. These characteristics present a significant barrier to potato improvement using classical breeding approaches [10]. Compared with tetraploids, diploid wild potato species have a rich genetic background, and it is easier to isolate drought resistant genes and analyze the molecular mechanisms of drought resistance, which is important for effectively improving drought resistance in the existing cultivated potato species. In a multi-year resource identification and evaluation study, we identified a diploid potato genotype, HS66, with strong drought tolerance, and a segregating F1 population was also obtained by crossing HS66 with the drought-sensitive diploid genotype CE125. After further evaluation of this population for two years, a highly drought-resistant genotype, P3-198, which showed higher resistance to drought than its parent HS66 was selected. P3-198 also maintains relatively high yield under drought stress, equivalent to that under normal irrigation. Polyethylene glycol (PEG) is a non-absorbable, non-metabolized and non-toxic osmotic agentthat has been widely used to induce osmotic stress in plants. The addition of PEG decreases the water potential of nutrient solutions, making less water available to the plant. It has been well established that PEG solutions are a more appropriate method than withholding water from soil-grown plants for simulating water deficit and for assessing the drought tolerance potential of plants [14]. Hence, we subjected P3-198 to PEG-induced drought stress and used RNA-seq to study genome-wide changes in gene expression to explore molecular mechanisms of drought resistance. The genes we identified in this study may be potential targets for breeding drought-resistant potato varieties.

## 2. Results

### 2.1. Transcriptome Sequencing, Assembly, and Mapping

**Figure 1:**
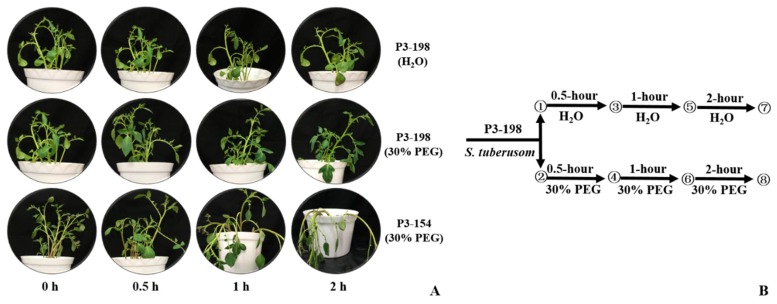
Phenotypes of P3-198 under polyethylene glycol (PEG) stress and well-watered conditions with P3-154 as the sensitive control. (**A**) P3-198 and P3-154 treated with 30% PEG or H_2_O (mock treatment) for 0, 0.5, 1, and 2 h. (**B**) The schematic showing the experimental design and the eight sampling points. Leaves were collected from three plants at each time point, and points ① and ② are the 0 h time points in P3-198.

A drought stress experiment was conducted by treating diploid potato genotype P3-198 seedlings with 30% PEG. Except for slight leaf rolling, which was observed after 2 h of drought stress, P3-198 looked similar to control seedlings treated with water while the drought sensitive genotype P3-154 wilted severely (Figure 1A). To gain a comprehensive overview of the transcriptional response of *S. tuberosum* to drought stress, we profiled the transcriptome of P3-198 treated with 30% PEG or H_2_O (mock treatment) for 0, 0.5, 1, and 2 h (Figure 1B).

Twenty-four libraries derived from seedling samples treated for 0.5, 1, and 2 h and the untreated samples (0 h), with three biological replicates per treatment, were sequenced using the Illumina HiSeqX10 system with the 150-cycle paired-end sequencing protocol. After data filtering and quality assessment, approximately 602 million paired end reads were produced, yielding an average of 25.1 million paired end reads (7.53 G clean bases) per sample. The GC percentage was 42.89%, and the Q30 percentage (sequencing error rate < 0.1%) was 91.71%. When mapping the RNA-seq reads to the DM reference genome, 78.51-85.90% of the reads could be mapped and 75.19-82.4% could be mapped uniquely to one location (Table S1).

### 2.2. Differential Gene Expression in Response to PEG Treatment

Fragments per kilobase of transcript per million mapped reads (FPKM) was used to represent the expression levels of genes that were generated using DESeq2. An *FDR* < 0.05 and a |log2 (fold change) | ≥1 were set as the threshold for significant differential expression. The DEGs were detected by performing pairwise comparisons at each time point within a single treatment. In total, 1699 and 2666 DEGs were detected for the H_2_O and PEG treatments, respectively. When comparing DEGs from the two treatments, 1665 genes were specifically differentially expressed between any two time points upon PEG treatment, 992 of which were up-regulated and 673 of which were down-regulated (Table 1).

**Figure 2:**
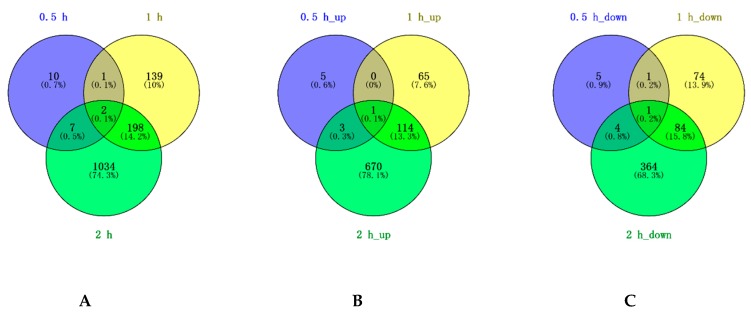
Venn diagram of differentially expressed genes (DEGs) under 0.5, 1 and 2 h of PEG stress in P3-198. (**A**), (**B**), and (**C**) show the Venn diagram results for the total, up-regulated, down-regulated genes, respectively.

A total of 20 (9 up-regulated and 11 down-regulated), 340 (180 up-regulated and 160 down-regulated), and 1241 DEGs (788 up-regulated and 453 down-regulated) were detected after 0.5, 1, and 2 h of PEG stress, respectively (Table 1, Figure 2). Venn diagram analysis showed that 10 (5 up-regulated and 5 down-regulated), 139 (65 up-regulated and 74 down-regulated), and 1034 DEGs (670 up-regulated and 364 down-regulated) were specifically regulated in P3-198 by 0.5, 1, and 2 h of PEG stress (Figure 2). A total of 198 DEGs (114 up-regulated and 84 down-regulated) were commonly regulated after 1 and 2 h, while only 1 DEG was commonly regulated after 0.5 and 1 h. A sharp increase in the number of DEGs after 1 and 2 h of stress indicated that an adaptive response to PEG stress was initiated in P3-198.

### 2.3. GO and KEGG Enrichment in DEGs

**Figure 3:**
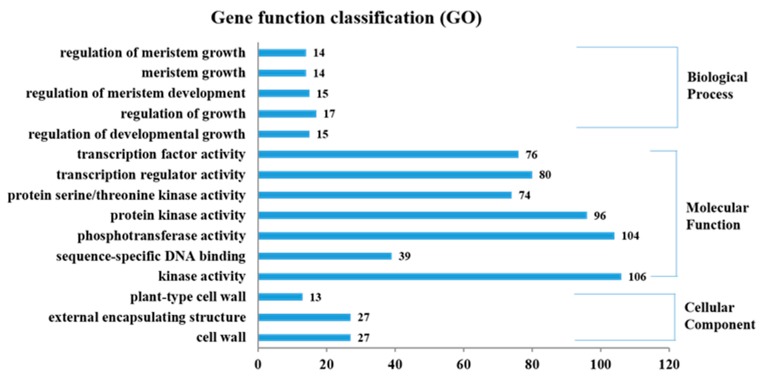
Gene ontology (GO) terms enriched in differentially expressed genes.

We next performed gene ontology (GO) analysis, and 1503 out of the 1665 DEGs were annotated to one or more GO terms and categorized into 718 functional groups. These functional groups were further divided into three categories: biological process, molecular function and cellular component (Figure 3). In the biological process class, regulation of meristem growth, meristem growth, regulation of meristem development, regulation of growth, and regulation of developmental growth were significantly enriched in DEGs (*FDR* < 0.05). In the molecular function class, a large number of DEGs were annotated to transcription factor activity, transcription regulator activity, protein serine/threonine, kinase activity, protein kinase activity, phosphotransferase activity, alcohol group as acceptor, sequence-specific DNA binding, and kinase activity, with most of the DEGs annotated to kinase activity (GO: 0016301; 106 DEGs) and phosphotransferase activity (GO: 0016773; 104 DEGs). In the cellular component category, the plant-type cell wall, external encapsulating structure, and cell wall were significantly enriched (Figure 3).

Out of 1503 annotated DEGs, 492 had Kyoto Encyclopedia of Genes and Genomes (KEGG) orthologs and were assigned to 95 pathways. The plant hormone signal transduction, flavonoid biosynthesis, glutathione metabolism, plant-pathogen interaction, galactose metabolism, and circadian rhythm pathways were significantly enriched in DEGs (*P*-value < 0.05). Metabolic pathways and biosynthesis of secondary metabolites had the highest number of DEGs (94 and 63, respectively; Table 2).

### 2.4. Differential Expression of TFs Regulated by PEG Treatment

**Figure 4:**
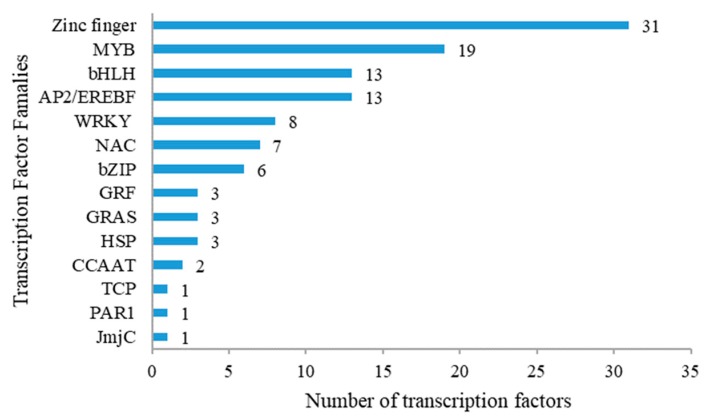
Transcription factors differentially expressed under drought stress in P3-198.

Among the 1665 DEGs differentially regulated by PEG stress, 124 encode TFs belonging to 12 families. Most TFs were ZFPs (31), followed by MYB (19), bHLH (13), AP2/EREBF (13), WRKY (8), NAC (7), and bZIP (6) (Figure 4, Table S2).

Among the 19 MYBs, there was an equal number of up- and down-regulated members, of which 8 genes were continuously up-regulated during stress, including *MYB3*, *MYB34*, and *MYB108*. The *MYB1R1* gene (PGSC0003DMG400001913), which was previously found to be involved in drought stress in potato [15], was significantly down-regulated. Among the bHLH, AP2/EREBF, and WRKY family members, most (over 75%) were up-regulated under drought treatment including *bHLH47*, *bHLH151*, *Upa20*, *WRKY2*, and *WRKY27*. The *bHLH130-like* (PGSC0003DMG400025263) gene had a high FPKM value (147.40) and *Upa20* (PGSC0003DMG400027042) expression increased more than 6-fold after 2 h of stress. Three AP2/EREBP genes, *AP2 domain-containing transcription factor 9* (PGSC0003DMG400012828), *Pti4* (PGSC0003DMG400016006), and *TSRF1* (PGSC0003DMG400017231), were significantly up-regulated at each stress time point. In addition, *NAC2* (PGSC0003DMG400002824), *NAC10* (PGSC0003DMG400009245), and *NAC domain-containing protein 21/22-like* (PGSC0003DMG400022134) were highly expressed under drought; *NAC2* was associated with biotic stress tolerance in a previous study [16]. Six bZIP TFs were responsive to drought, with two genes PGSC0003DMG400008011 and PGSC0003DMG400022931, encoding ABF and BZIP1, respectively, very highly up-regulated under drought stress. The FPKM value of the ABF TF gene was high (196.16) at the 2 h stress time point.

Based on the individual finger structures and spacing, the 31 ZFPs are further divided into different classes: C2H2, RING finger, CCT, CCCH, CHP, HD, B-box, Dof, GDSL-like Lipase/Acyl hydrolase, GATA, and AN1. There was an equal number of up- and down-regulated members (Figure 4, Table S2). The *GATA21* (PGSC0003DMG400000280) gene was down-regulated at all time points after the application of stress. One Dof gene (PGSC0003DMG400019528) had the highest FPKM value (133.78) at the 2 h stress time point. Two AN1 domain genes PGSC0003DMG400002697 and PGSC0003DMG400027241, which encode stress-associated protein 8 (SAP8) and SAP16-like, respectively, were significantly up-regulated after 2 h of PEG treatment (Figure 4).

### 2.5. Protein Kinases Differentially Expressed in Response to Drought Stress

**Figure 5:**
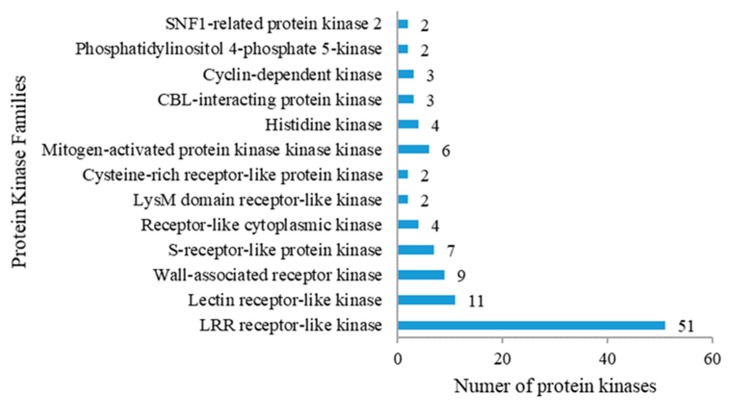
Protein kinases differentially expressed under drought stress in P3-198.

Protein kinases (PKs) act as signal transducer/receptor proteins in membranes and play a crucial role in phosphorylation events. A total of 139 PK genes were differentially expressed, and most of them (84) encode receptor-like protein kinases (RLKs), with more than half (60.7%) encoding leucine-rich repeat receptor-like protein kinases (LRR-RLKs) (Figure 5, Table S3). Under drought stress, the LRR-RLKs *BRASSINOSTEROID INSENSITIVE 1 (BRI1)-ASSOCIATED RECEPTOR KINASE 1* (*BAK1*, PGSC0003DMG400012594) and *SUPPRESSOR OF BIR1* (*SOBIR1*, PGSC0003DMG400015157) were up-regulated and *FLAGELLIN SENSITIVE 2* (FLS2, PGSC0003DMG400008296) was down-regulated.

Genes that encode the other RLKs, lectin RLKs (LecRLKs, 11 members), wall-associated receptor kinases (WAKs, 9 members), S-receptor-like protein kinases (SPKs, 7 members), receptor-like cytoplasmic kinases (RLCKs, 4 members), lysM domain receptor-like kinases (LYKs, 2 members), and cysteine-rich receptor-like protein kinases (CRKs, 2 members), were differentially expressed in response to drought stress. All nine WAK genes were up-regulated at all stress time points. *CRK3* (PGSC0003DMG400013525), *CRK42* (PGSC0003DMG402000515), and one RLCK gene, *Pto* (PGSC0003DMG400037460), were induced by PEG-stress.

The other PKs identified under PEG stress belong to the following families: MAPKKK, histidine kinases (HKs), CBL-interacting protein kinases (CIPKs), cyclin-dependent kinases (CDKs), and sucrose non-fermenting 1-related protein kinase (SnRK2) (Figure 5; Table S3). Three MAPKKK genes (PGSC0003DMG400004036, PGSC0003DMG400005773, PGSC0003DMG400015021) were dramatically up-regulated by drought stress, and the expression of *CIPK11* (PGSC0003DMG400030462), *CIPK13* (PGSC0003DMG400011641), *CIPK16* (PGSC0003DMG400028479), three CDK genes and two SnRK2 genes was found to gradually increasewith increasing duration of PEG stress.

### 2.6. Stress-Related Protein Genes Induced by Drought

#### 2.6.1. Genes Encoding Redox Regulation-Related Proteins

A set of 24 genes related to redox regulation were differentially expressed in P3-198 under drought stress. These genes include those encoding ascorbate peroxidase (APX), peroxidase (POD), oxidoreductase, monodehydroascorbate reductase (MADR), NADPH: protochlorophyllide, Acyl-CoA syntheses, glutathione S-transferase (GST), and acyl CoA reductase, showing that redox regulation is a potential molecular mechanism of drought stress resistance (Table S4).

#### 2.6.2. Genes Related to Carbohydrate Metabolism and Osmotic Adjustment

**Figure 6:**
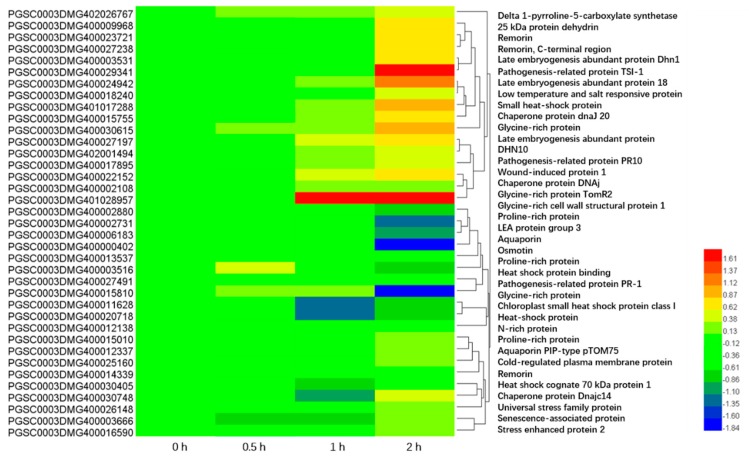
Genes coding for osmotic adjustment proteins differentially expressed under drought stress.

Eight genes encoding UDP-glucosyltransferase (UGT) family proteins and 40 genes encoding osmotic regulatory proteins were highly differentially expressed under PEG-induced drought stress. These proteins include dehydrins, proline-rich proteins (PRPs), late embryogenesis abundant proteins (LEAs), aquaporins (AQPs), osmotins, heat shock proteins (HSPs), chaperone proteins, pathogenesis-related proteins (PRs), asparagine-rich proteins (N-rich protein, NRPs), and low temperature and salt responsive proteins (Figure 6). Most of these genes were up-regulated. Among these, PGSC0003DMG400009968, PGSC0003DMG400027197, and PGSC0003DMG400002880, which encode dehydrin, LEA, and PRP proteins, respectively, were the most highly expressed with FPKM values greater than 1000. We also found one DEG encoding 1-pyrroline-5-carboxylate synthetase (P5CS), a key enzyme in the biosynthesis of proline. The expression of the P5CS gene increased gradually with extended duration of PEG stress (Figure 6).

### 2.7. Validation of Differential Gene Expression

**Figure 7:**
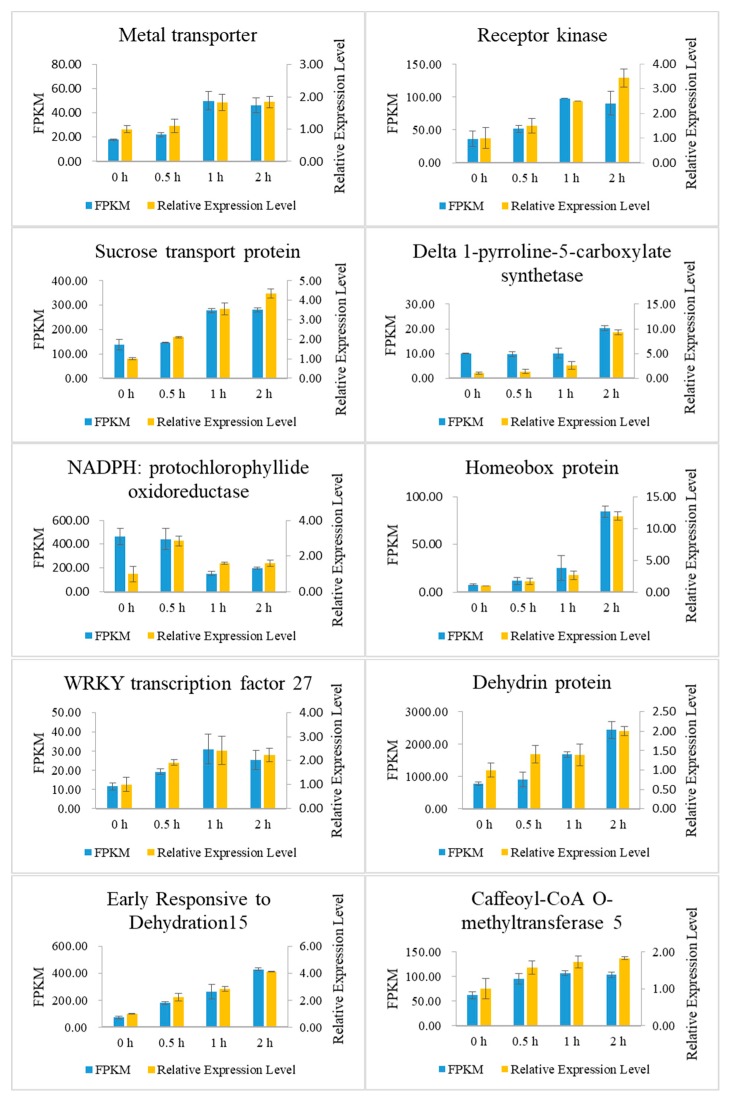
Quantitative real-time PCR analysis of differentially expressed genes.

To validate the differential expression results from transcriptome sequence data analysis, the expression of 10 randomly selected DEGs in P3-198 under PEG-induced drought stress and control conditions was evaluated by qRT-PCR. These genes encode a metal transporter, a receptor kinase, a P5CS, a WRKY TF 27, a NADPH, and a dehydrin protein (Figure 7, Table S5). The expression levels determined by qRT-PCR were in agreement with the changes in transcript abundance determined by RNA-seq analysis, which suggests that our transcriptome profiling data are highly reliable (Figure 7).

## 3. Discussion

### 3.1. The Diploid Potato Genotype P3-198 is an Excellent Drought-Resistant Material

Potato cultivars are highly heterozygous autotetraploids. In long-term potato breeding programs, many germplasm resources with similar genetic backgrounds were used, resulting in potato cultivars that have a narrow genetic background and close kinship. Diploid wild potato species often have strong resistance to stress, and these species are important drought-resistant resources. Hence, further research to reveal the drought-resistance mechanisms in diploid wild potato species is of great significance for breeding drought-resistant potatoes. The diploid potato genotype P3-198 was obtained from screening a segregating F1 population derived from a cross between the highly drought-resistant material HS66 and drought-sensitive material CE125. When grown under drought conditions for two consecutive years, P3-198 maintained excellent growth and had a yield equivalent to that of P3-198 plants grown under normal irrigation conditions. In this study, P3-198 was subjected to PEG-induced drought stress, and a large number of TFs, PKs, and proteins associated with drought resistance were differentially expressed, indicating that P3-198 has a strong drought response.

### 3.2. The Regulatory Role of Transcription Factors in Drought Resistance

Transcription factors (TFs) play an essential role in many different signal transduction pathways in plants during stress by activating the expression of downstream target genes through specific binding to *cis*-acting elements [17]. MYB/MYC, bZIP, AP2/EREBP, WRKY, DREB (AP2/ERF) and NAC are well-known TF families that are responsive to drought [18]. Also, MYB, NAC and bZIP TFs activate the expression of ABA-responsive genes under drought stress [19,20]. In this study, 124 (7.4%) of the 1665 genes differentially regulated under PEG stress encode TFs. These TFs mainly belong to the ZFP, MYB, bZIP, bHLH, AP2/ERF, WRKY, and NAC families. Among the TFs identified as being differentially expressed in the present study, several are known to play a role in drought resistance. Overexpression of potato *StMYB1R-1*, which was up-regulated by PEG stress in P3-138, was reported to induce the expression of *AtHB-7*, the water channel protein gene *RD28*, aldehyde dehydrogenase (*ALDH*), and early response to dehydration 15 (*ERD15*), finally leading to increased drought resistance [15]. Another MYB TF gene, *MYB108*, which was down-regulated by PEG stress in P3-198, was reported to be involved in the response to drought stress in *Arabidopsis* [21].

AREB/ABF bZIP TFs bind to the ABRE, which is the major *cis*-acting regulatory element regulating ABA-responsive gene expression. The potato bZIP TF *StABF1* is induced in response to drought, ABA, salt, and cold stress [22]. Another bZIP TF, *AtbZIP1*, is induced by salt and low temperature stress and is regulated by ABA signal transduction [23]. In this study, two genes encoding AREB/ABF bZIP TFs, *ABRE binding factor X1* and *BZIP1*, were significantly up-regulated in response to PEG-induced drought stress, which suggests they might be regulators of ABA-dependent stress signaling pathways in P3-198.

Ethylene response factor (ERF) TFs contain a conserved AP2⁄ERF DNA-binding domain [24] and have been reported to regulate plant development and responses to biotic/abiotic stimuli including drought, salt and cold. The tomato ERF *TSRF1* (or *TERF1*) positively regulates pathogen resistance in tomato and tobacco and improves tolerance to osmotic and drought stress in monocot rice [25,26]. In this study, 13 DEGs with highly up-regulated expression under PEG stress encode proteins homologous to *TSRF1*, suggesting that these proteins positively regulate the response to drought stress in P3-198.

NAC TFs are specifically found in plants and play important roles in the response to drought, high salt, low temperature and hormones. Some drought-responsive NAC TFs have been identified in rice and *Arabidopsis*, including *OsNAC5*, *OsNAC10*, *SNAC1/2*, *ONAC045*, *ANAC019*, *ANAC055*, and *ANAC072* (*RD26*) [27,28]. Seven NAC TFs with up-regulated expression under drought were identified in this study, including *NAC2* and *NAC10*. *StNAC2* has been shown to be involved in signal transduction cascades activated in response to *Phytophthora infestans*, wounding, salt, and drought stress in potato [16]. Overexpression of *StNAC2* in transgenic potato significantly enhanced salt tolerance in vitro and drought tolerance in soil-grown plants [29].

### 3.3. Protein Kinases May Play an Important Role in the Response to PEG-Induced Drought Stress in P3-198

PKs play an important role in the response to plant drought and other abiotic stresses by activating signal transduction pathways. These PKs include RLKs, MAPKs and calcium/calmodulin-dependent protein kinases (CDPKs) [30]. In this study, 60.4% of differentially expressed PKs encoded RLKs. One of these RLKs, FLS2 activates the plant immune response by binding to the pathogen flagellin protein and also plays a role in ABA-induced stomatal closure through intermolecular interactions with BAK1, a co-receptor of flg22 [31,32]. The differential expression of RLKs in this study, suggests that different membrane receptor recognition pathways are activated by PEG stress.

Ca^2+^ is a ubiquitous second messenger that is involved in signaling pathways and is induced by a variety of environmental and developmental stimuli. In response to these stimuli, cells generate transient changes in the intracellular Ca^2+^ concentration, and these changes are sensed and decoded by Ca^2+^ sensors. The major Ca^2+^ sensors are calmodulins, calmodulin-like proteins (CMLs), CBLs, CDPKs, and CIPKs [33]. CDPKs are the main sensors in stress signaling pathways, and act by modulating ABA signaling and reducing the accumulation of ROS [34,35]. Members of Ca^2+^ sensor families in *Arabidopsis* and rice have been reported to regulate ROS production during immune signaling and to be involved in drought stress response; these PKs include AtCPK6, AtCPK8, AtCPK10, AtCPK23, CML37, and CML42 [36,37,38,39]. Seven of the DEGs in this study code for Ca^2+^ sensors (CML3, CML30, CML36, CBL1, CNL7, CIPK13, and CIPK16), and all except CML3 were induced by PEG stress, suggesting that Ca^2+^ sensors play an important positive regulatory role in the response to PEG-induced drought in P3-198.

SnRK2 is a PK that is only present in plants. SnRK2 expression is induced by ABA, dehydration, and high salt stress, and it participates in ABA signal transduction. Eight *StSnRK2* genes (*StSnRK2.1*- *StSnRK2.8*) were cloned from the potato cultivar, Longshu 3, and the promoters of these genes contained the drought stress-related cis elements LTRE, DREB, and ABRE [40,41]. In this study, two DEGs encoding SnRK2 were induced by PEG stress, which indicates that SnRK2 genes might positively regulate the drought response in potato genotype P3-198.

Reversible protein phosphorylation controlled by PKs is important for cell recognition and signal transduction, and protein phosphatases are important regulators of reversible protein phosphorylation. Type 2C protein phosphatases (PP2Cs) represent the major group of protein phosphatases in plants and play important roles in the response to drought, cold and ABA [42]. In this study, four DEGs were found to encode PP2C proteins, with three genes significantly up-regulated under PEG stress. These genes may positively regulate the response to drought in P3-198.

### 3.4. Stress-Related Proteins Protect the Plant Cell from Stress Damage

In this study genes related to redox regulation, carbohydrate metabolism, and osmotic adjustment were differentially regulated in response to PEG stress. These genes are assumed to be involved in stress resistance. For example, UDP-glycosyltransferases, which catalyze glycosylation and are required for a number of plant growth and developmental processes, are also involved in abiotic stress adaptation [43]. A UDP-glycosyltransferase74B1-like gene was shown to be up-regulated under drought stress in tobacco roots [44] and *Arabidopsis* uridine diphosphate (UDP)-glycosyltransferase 76C2 was down-regulated by ABA and water deficit conditions [45]. Glutathione S-transferases (GSTs) play roles in cellular detoxification and stress tolerance, and in potato the *StGST* genes are mainly repressed in response to abiotic stresses and induced in response to biotic stress [46]. Seven of eight GST genes were up-regulated in the present study, indicating that these genes may play positive roles in drought resistance in P3-198.

Under drought or salt stress, the accumulation of small molecular organic compounds that stabilize biological structures can directly protect plant cells from damage. Genes encoding proteins or enzymes involved in the synthesis of small molecules were identified in this study. These proteins mainly include proline-rich protein (PRP), trehalose-6-phosphate synthase (TPS), water channel proteins, chaperone proteins, LEA protein, and dehydrin protein [47,48]. Proline is one of the most important osmoprotectants. Under stress, proline protects membranes from damage and stabilizes protein structures [49]. The PRP gene *StGCPRP* is highly expressed in guard cells of potato but is negatively regulated by drought stress [50]. Two PRP genes were also found to be down-regulated with extended duration of stress in our study, indicating the negative regulatory roles of these genes in drought stress. TPS is the key enzyme in trehalose synthesis. TPS genes expression has been shown to improve drought tolerance in various plants, including tobacco, cotton, maize, and potato [51,52]. Consistent with their role in stress tolerance, *StTPS* genes were found to contain abiotic stress-responsive elements [48]. Two TPS genes were up-regulated under PEG stress in P3-198, indicating they play a positive role in protection against drought damage. LEA proteins play crucial roles in protecting cells from dehydration [53]. Among the 1665 DEGs, the highest expression levels were observed for genes encoding a dehydrin protein and a LEA protein (FPKM values of 2497.18 and 1898.12, respectively, after 2 h of stress), indicating the important roles of these genes in protecting P3-198 from damage during drought stress.

### 3.5. The Potential Molecular Mechanisms Underlying the Response to Drought Stress in P3-198

In the absence of stress, ABA is maintained at low levels in plant cells to maintain normal growth, but in the presence of stress such as drought, the level of ABA can increase dramatically. In this study a gene (PGSC0003DMG400004020) encoding zeaxanthin epoxidase (ZEP), a key enzyme involved in ABA biosynthesis and plant tolerance to drought [54], was up-regulated at all time points under PEG stress. Overexpression of ZEP genes were found to increase drought and salt stress tolerance in *Arabidopsis* and tobacco [55], so the up-regulation of PGSC0003DMG400004020 in P3-198 indicates that it has a positive role in drought resistance.

**Figure 8:**
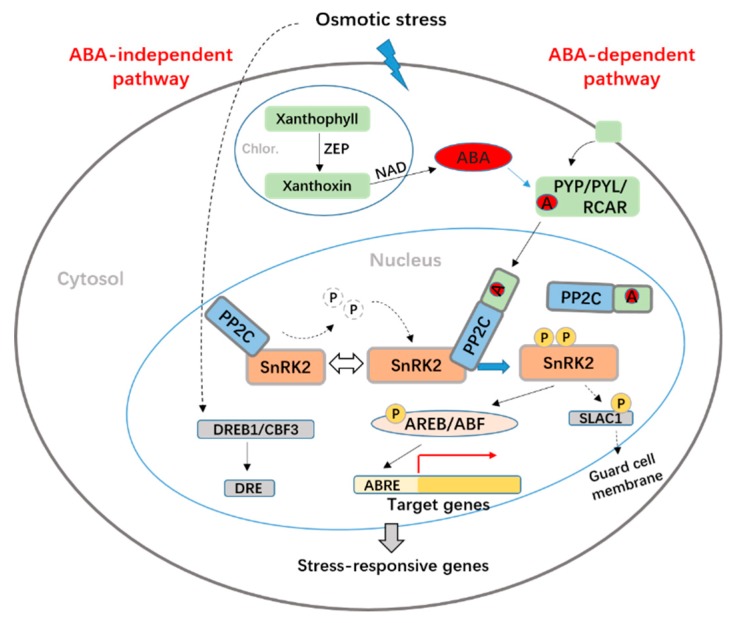
Possible molecular mechanisms involved in drought response in potato P3-198.

The ABA-dependent signal transduction pathway consists of four core components, PYR/PYL/RCAR, PP2C, SnRK2, and ABF/AREB, that regulate the response to stress [56]. The PYR/PYL/RCAR receptors perceive ABA, and the resulting complex inactivates downstream PP2C, which activates SnRK2 [57]. SnRK2 can then phosphorylate downstream TFs, which inhibit stomatal opening by regulating gene transcription, allowing the plant to adapt to stress. An ABA receptor PYL4 gene (PGSC0003DMG400011033) was up-regulated in this study, indicating that it has an important role in recognizing ABA and activating the drought stress response. PP2Cs negatively regulate ABA responses by keeping SnRK2 kinases in an inactive state in the absence of ABA [42]. We identified four DEGs related to PP2Cs. Of these, one gene encoded by PGSC0003DMG400011273 was significantly down-regulated during exposure to drought stress. We also identified two SnRK2 genes, which showed significant increases in expression in P3-198 during drought stress. As mentioned above, one ABF TF gene was significantly up-regulated in response to PEG-induced drought stress. This TF could bind to the downstream cis-acting regulatory element ABRE and regulate ABA-responsive gene expression under stress in P3-198. MYBR, another TF functioning in the ABA-dependent signaling pathway, was also found to be up-regulated in this study with FPKM values of 20.73, 17.49,24.39, and 48.82 after 0, 0.5, 1, and 2 h of stress, respectively. We also found an up-regulated ABA-independent DREB TF gene in our RNA-seq dataset, but the expression of this gene was very low (FPKM values of only 4.32, 6.46, 2.27, and 4.41 after 0, 0.5, 1, and 2 h of PEG stress, respectively). In contrast, the FPKM values of the ABF gene at the same time points were very high (36.65, 43.71, 75.07, and 196.16 after 0, 0.5, 1, and 2 h of stress, respectively). These results suggest that the ABA-dependent signaling pathway may play a more important role than the ABA-independent signaling pathway in P3-198 (Figure 8).

Though we could not dissect the complete drought resistance mechanism, the drought resistance gene expression dataset generated in this study provides a resource for isolating drought resistance genes and analyzing the molecular mechanisms of drought resistance in potato. A comparative study of the drought response in soil-grown plants of P3-198 will be done in our future study, which would be very informative.

## 4. Materials and Methods

### 4.1. Plant Materials

The drought-resistant genotype P3-198 was identified in the F1 progeny of a cross between the diploid drought-tolerant parent HS66 and drought-sensitive parent CE125. HS66 was derived from the cross between common cultivar dihaploid and *S. phureja*. The CE125 was derived from a cross between two diploid potato clones USW5337.3 (C) and 772102.37 (E), with genetic background of *S. phureja* and *S. vernei*, respectively [58,59]. During the assessment of the drought tolerance of 130 individuals in the F1 generation for two consecutive years, it was found that P3-198 exhibited excellent growth and strong drought resistance, and there was no significant difference in yield under drought stress (0.36, 0.39 kg per plant, respectively) and control conditions (0.37, 0.41 kg per plant, respectively) for two consecutive years. Further study of P3-198 under PEG-induced drought stress also confirmed that P3-198 is highly resistant to drought.

### 4.2. PEG-Induced Drought Stress

The seeds were sown in nutritional pots (18 cm × 13 cm × 12 cm) filled with vermiculite and charcoal and maintained in a greenhouse under natural lighting with day temperatures of 16–20 ℃ and night temperatures of 8–10 ℃. After 3-4 leaves had developed, seedlings of similar size were selected and transplanted into pots (19 cm × 14 cm × 13 cm) containing perlite without holes in the bottom. After 2 days of slow growth, a stress treatment with 30% PEG was performed. The 30% PEG nutrient solution was prepared using PEG 8000 (AMRESCO). The solution was placed in a water bath preset at 30 ℃ to allow the PEG 8000 to rapidly dissolve. The solution was continuously stirred with a glass rod for half an hour, then 500 mL was added to each pot. Seedlings receiving the same volume of water were used as the control. There were three replicates for each treatment. At 0, 0.5, 1, and 2 hours of treatment, the leaf samples were harvested from three different plants for each treatment, immediately frozen in liquid nitrogen, and stored at −80 ℃ for RNA extraction and sequencing.

### 4.3. RNA Preparation

Total RNA was extracted from the plant leaves using TRIzol reagent (Invitrogen, Carlsbad, CA, USA). Sequencing libraries were generated using the NEBNext UltraTM RNA Library Prep Kit for Illumina (NEB, Ipswich, MA, USA) following the manufacturer’s recommendations. The quality and size of the libraries were assessed on the Agilent 2200 TapeStation system (Agilent, Santa Clara, CA, USA) prior to sequencing. Then, the libraries were sequenced on the Illumina HiSeqX10 platform, and paired-end reads were generated with the 150-cycle paired-end sequencing protocol.

### 4.4. Data Quality Control

Raw data in FASTQ format from the current trial are available from the Genome Sequence Archive (GSA) under accession CRA001283. Clean data (clean reads) were obtained by removing reads containing adapter sequence, reads containing poly-N and low-quality reads from the raw data. Q20, Q30, GC-content, and sequence duplication level were then calculated for the clean data. All downstream analyses were performed using high-quality clean data.

### 4.5. Mapping Reads to the Genome

The clean reads were mapped to the reference genome sequence using HISAT2 tools [60]. The genome sequence (SolTub 3.0) of the reference accession, the doubled haploid *S. tuberosum* Group *Phureja* clone DM1-3 516R44 (referred to as DM), and annotation files were downloaded from the ENSEMBL plants database (ftp://ftp.ensemblgenomes.org/pub/plants/release-34/fasta/solanum_tuberosum/dna/) [61]. Only reads with a perfect match or one mismatch were further analyzed and annotated based on the reference genome. The transcription units (TUs) and gene transfer format (GTF) files were assembled using StringTie [62]. Gene expression levels were estimated by calculating fragments per kilobase of transcript per million fragments mapped (FPKM) and normalized using HTseq-count and DESeq2 [63,64].

### 4.6. Differential Expression Analysis

Differential expression analysis was performed using DESeq2 based on the negative binomial distribution [65]. The resulting *P*-values were adjusted using the Benjamini and Hochberg’s approach for controlling the false discovery rate (FDR). Genes with a normalized expression fold-change greater than 2 and an adjusted *P*-value < 0.05 were defined as differentially expressed. Differential expression was determined by comparing the expression levels at different time points within a single treatment. Then, the differentially expressed genes (DEGs) from the PEG samples were compared with the DEGs from the H_2_O samples. The DEGs were annotated based on the functional annotation information of genes in the SolTub_3.0 version of the *S. tuberosum* genome from ENSEMBL and the annotations of genes orthologous to potato in *Arabidopsis*.

### 4.7. GO and KEGG Enrichment

Gene ontology (GO) enrichment analysis of the DEGs was performed using the GOseq R package based on the Wallenius non-central hyper-geometric distribution [66], which adjusts for gene length bias. KOBAS software was used to test for statistically significant enrichment of DEGs in KEGG pathways [67]. All annotated *S. tuberosum* genes in the SolTub_3.0 assembly in ENSEMBL were used as the background for GO and KEGG enrichment analysis. GO and KEGG terms with a corrected *P*-value < 0.05 were considered significantly enriched for the DEGs.

### 4.8. Quantitative Real-Time PCR (qRT-PCR) Validation

Ten DEGs were randomly selected for qRT-PCR to verify the RNA-seq results (Table S5). The RNA samples were the same as those used for the RNA-seq analysis. For cDNA synthesis, 1–2 µg of total RNA was reverse transcribed in a 20 µL reaction using Superscript II reverse transcriptase (Invitrogen, Carlsbad, CA, USA). qRT-PCR was performed in a 20 µL reaction mixture with 10 µL SYBR^®^ Green Supermix (Cat. No.170-8882, BioRad, Hercules, CA, USA), 0.5 µL each of the forward and reverse primers, 7 µL of double-distilled H_2_O and 2 µL (40 ng/µL) of the cDNA. All reactions were performed in triplicate for each cDNA sample with an annealing temperature of 60 ℃ and a total of 40 cycles of amplification. The relative expression level of each gene was calculated using the 2^−∆∆Ct^ method [68] with expression normalized against the internal reference gene GAPDH and with expression at 0 h used as the control. The primers used in this study were designed using Primer 5 and are listed in Table S5.

## Figures and Tables

**Table 1:** Differentially expressed genes that were specifically up-regulated or down-regulated by PEG drought stress in potato genotype P3-198.

Comparison	Number of DEGs	Up-Regulated DEGs	Down-Regulated DEGs
0.5 vs 0 h	20	9	11
1 vs 0 h	340	180	160
2 vs 0 h	1241	788	453
1 vs 0.5 h	102	42	60
2 vs 0.5 h	773	530	243
2 vs 1 h	270	234	36
Total *	1665	992	673

* This is the total number of genes specifically differentially expressed in response to PEG stress after removing duplicate genes by performing pair-wise comparisons.

**Table 2:** Main Kyoto Encyclopedia of Genes and Genomes (KEGG) pathways enriched in differentially expressed genes in P3-198.

Pathway Terms	ID	DEGs Number	*P*-Value
Plant hormone signal transduction	sot04075	29	0.0011
Flavonoid biosynthesis	sot00941	9	0.0039
Glutathione metabolism	sot00480	11	0.0199
Plant-pathogen interaction	sot04626	17	0.0303
Galactose metabolism	sot00052	7	0.0317
Circadian rhythm plant	sot04712	6	0.0386
Metabolic pathways	sot01100	94	0.8165
Biosynthesis of secondary metabolites	sot01110	63	0.3421
Phenylpropanoid biosynthesis	sot00940	15	0.1741

## Supplementary Materials

Supplementary materials can be found at http://www.mdpi.com/1422-0067/20/4/852/s1.
