# Supplementary material for: Transcriptome Profiling Reveals Effects of Drought Stress on Gene Expression in Diploid Potato Genotype P3-198
Source: Int J Mol Sci. 2019 Feb 15;20(4):852. doi: 10.3390/ijms20040852 (PMC6413097; doi:10.3390/ijms20040852)
Supplement: Supplementary file 1 [file ijms-20-00852-s001.pdf]

Table S1. Basic summary of RNA-sequencing results.

Samples	Total reads	Total bases (G)	Mapped reads	Unique mapped reads
H_2O_0h_rep1	27,390,958	8.22	22,543,455 (82.30%)	21,669,975 (79.11%)
H2O_0h_rep2	24,015,524	7.20	19,846,371 (82.64%)	19,024,798 (79.22%)
H2O-0h_rep3	22,064,474	6.62	18,953,986 (85.90%)	18,180,512 (82.40%)
H2O-0.5h_rep1	21,485,547	6.45	17,577,376 (81.81%)	16,867,536 (78.51%)
H2O-0.5h_rep2	21,861,706	6.56	18,246,281 (83.46%)	17,541,679 (80.24%)
H2O-0.5h_rep3	23,129,046	6.94	19,002,760 (82.16%)	18,233,250 (78.83%)
H2O-1h_rep1	21,278,804	6.38	18,169,646 (85.39%)	17,437,650 (81.95%)
H2O-1h_rep2	23,851,958	7.16	19,643,588 (82.36%)	18,835,824 (78.97%)
H2O-1h_rep3	23,261,356	6.98	19,150,913 (82.33%)	18,401,103 (79.11%)
H2O-2h_rep1	23,801,719	7.14	19,482,815 (81.85%)	18,717,037 (78.64%)
H2O-2h_rep2	27,968,144	8.39	23,952,815 (85.64%)	22,908,967 (81.91%)
H2O-2h_rep3	25,599,773	7.68	21,026,359 (82.13%)	20,078,257 (78.43%)
PEG-0h_rep1	25,378,571	7.61	21,592,176 (85.08%)	20,778,213 (81.87%)
PEG-0h _rep2	27,461,776	8.24	22,508,859 (81.96%)	21,667,287 (78.90%)
PEG-0h_rep3	21,329,412	6.40	18,240,320 (85.52%)	17,483,542 (81.97%)
PEG-0.5h_rep1	28,153,931	8.45	22,999,471 (81.69%)	220,86,924 (78.45%)
PEG-0.5h_rep2	25,438,148	7.63	19,971,358 (78.51%)	19,127,255 (75.19%)
PEG-0.5h_rep3	28,715,876	8.61	23,602,458 (82.19%)	22,609,075 (78.73%)
PEG-1h_rep1	30,024,679	9.01	25,607,964 (85.29%)	24,575,892 (81.85%)
PEG-1h_rep2	26,414,784	7.92	21,408,401 (81.05%)	20,517,217 (77.67%)
PEG-1h_rep3	29,580,178	8.87	25,056,568 (84.71%)	24,050,366 (81.31%)
PEG-2h_rep1	27,126,999	8.14	22,202,260 (81.85%)	21,205,564 (78.17%)
PEG-2h_rep2	20,784,648	6.24	17,778,336 (85.54%)	17,068,332 (82.12%)
PEG-2h_rep3	26,254,441	7.88	21,456,566 (81.73%)	20,591,615 (78.43%)

Table S2. Transcription factors differentially expressed under PEG treatment.

TF families	Numbers	Types/Known genes*
Zinc finger	31	C2H2, C3HC4 (RING) type, C2H2L, HD, B-box, Dof, CCT, GDSL-like Lipase/Acylhydrolase, CHP-rich, GATA, CCCH, A20/AN1, AN1/C2H2
MYB	19	MYB1R1, MYB3, MYB34, MYB92, MYB108-1, AS1, DIV2B, DIV3B, RL3
bHLH	13	bHLH151, Upa20, BEE3
AP2/EREBF	13	TSRF1, ERF054, ERF061, Pti4
WRKY	8	WRKY2, WRKY27
NAC	7	NAC2, NAC10
bZIP	6	ABRE binding factor (ABF), bZIP1
GRF	3	GRF10
GRAS	3	GRAS4
HSP	3	HSFA-1, HSFA9
CCAAT	2	CBF/NF-YA3, CBF/NF-YB
TCP	1	TCP20-like
PAR1	1	PAR1
JmjC	1	hypoxia-inducible factor 1-alpha inhibitor

*: here only listed known genes or gene types.

Table S3. Protein kinase genes differentially expressed under drought stress.

Protein kinase families		Numbers	Known genes*
Receptor-like protein kinase, RLK	leucine-rich repeat receptor-like kinases, LRR-RLK	51	FLS2, SR160, BRI1, BAK1, CLAVATA1
	Lectin receptor-like kinases, LecRLK	11	--
	wall-associated receptor kinase, WAK	9	--
	S-receptor-like protein kinase, SPK	7	S6, SD2-5
	receptor-like cytoplasmic kinases, RLCK	4	Pto, PBS1, CRCK2
	lysM domain receptor-like kinase	2	LYK4
	cysteine-rich receptor-like protein kinase, CRK	2	CRK3, CRK42
Mitogen-activated protein kinase kinase kinase, MAPKKK		6	--
Histidine kinase, HK		4	--
CBL-interacting protein kinase, CIPK		3	ACRE216, CIPK13, CIPK16
Cyclin-dependent kinases, CDK		3	--
Phosphatidylinositol 4-phosphate 5-kinase, P4P5K		2	--
Sucrose non-fermenting 1 (SNF1) -related protein kinase 2, SnRK2		2	SNF4

--: no known genes. *: here only listed known genes.

Table S4. Differentially expression genes encoding redox regulation-related proteins under drought stress.

Gene ID	*Ath* ID	FPKM_0 h	FPKM_0.5 h	FPKM_1 h	FPKM_2 h	Description
PGSC0003DMG400003645	--	6.43	7.14	4.30	3.34	Ascorbate peroxidase
PGSC0003DMG400002703	AT3G52880	99.98	110.04	136.63	202.78	Monodehydroascorbate reductase
PGSC0003DMG400016223	AT2G22420	0.94	0.87	1.36	3.71	Peroxidase 17
PGSC0003DMG400022341	AT5G19890	0.81	1.05	0.82	2.04	Suberization-associated anionic peroxidase 2
PGSC0003DMG400022342	--	72.32	62.74	89.14	126.95	Suberization-associated anionic peroxidase
PGSC0003DMG400024285	AT4G26010	69.98	78.96	33.78	35.72	Peroxidase 44
PGSC0003DMG400032199	--	1.58	1.04	1.51	4.46	Peroxidase
PGSC0003DMG400004822	AT4G17370	5.85	6.18	12.16	11.03	Oxidoreductase
PGSC0003DMG400006940	--	6.57	6.74	3.26	2.84	Oxidoreductase
PGSC0003DMG400011098	--	1.46	0.79	0.36	1.84	Oxidoreductase
PGSC0003DMG400011834	--	2.45	2.39	1.56	0.88	Oxidoreductase
PGSC0003DMG400019973	AT2G46890	1.77	1.72	1.19	0.67	Oxidoreductase
PGSC0003DMG400025962	--	2.64	2.12	1.38	1.01	Oxidoreductase
PGSC0003DMG400032208	--	15.17	16.41	3.58	7.73	Oxidoreductase, 2OG-Fe(II) oxygenase family protein
PGSC0003DMG400015356	AT4G27440	428.02	459.05	197.09	266.21	NADPH:protochlorophyllide oxidoreductase
PGSC0003DMG400025007	AT4G27440	412.04	396.46	145.39	166.97	NADPH:protochlorophyllide oxidoreductase
PGSC0003DMG400007786	AT1G78380	2.36	3.22	4.82	5.27	2,4-D inducible glutathione S-transferase
PGSC0003DMG400016722	AT1G02920	38.00	38.17	6.36	10.04	Glutathione S-transferase
PGSC0003DMG400019728	AT1G65820	53.81	61.59	81.99	104.27	Microsomal glutathione s-transferase
PGSC0003DMG400031094	AT1G78380	0.62	1.02	0.53	1.71	Glutathione S-transferase/peroxidase
PGSC0003DMG400007405	AT5G22500	7.80	6.78	9.33	27.60	Acyl CoA reductase
PGSC0003DMG400011601	--	2.90	5.04	4.00	6.99	2,4-dienoyl-CoA reductase
PGSC0003DMG400033642	--	11.87	10.81	24.10	29.07	2,4-dienoyl-CoA reductase
PGSC0003DMG400014345	AT2G29550	151.71	149.58	202.90	292.49	Acyl-CoA syntheses

Ath, Arabidopsis thaliana; FPKM, Fragments per kilobase of exon per million fragments mapped; -- means non-significant orthologous in *Arabidopsis*.

Table S5. Annotations and qRT-PCR primers for 10 randomly selected DEGs.

Gene ID	Gene annotation	Forward primer (5' - 3')	Reverse primer (5' - 3')
PGSC0003DMG400013108	Metal transporter	TGGCAGAACATCCGTGG	GACTCCGAATCAGACAAACC
PGSC0003DMG400015157	Receptor kinase	GCCTGCCGTGAGTCAAGTT	CAGAGAAGACCAACGCCGAT
PGSC0003DMG402026767	Delta 1-pyrroline-5-carboxylate synthetase	GTAACGTCTCACAGCTCCCA	TCTGACCTACAGCCGCAC
PGSC0003DMG400009213	Sucrose transport protein	AACCAAAGCCTGCGACAT	GCTCGTTTTCCCGGACT
PGSC0003DMG400025007	NADPH: protochlorophyllide oxidoreductase	CCACCACTGACTAGAACGAC	TAAACCTCCCGCCATAC
PGSC0003DMG400017640	Homeobox protein	GGTTCATCCATAGGCACG	GATTGTTGAAAGACCAGTAG
PGSC0003DMG401033880	WRKY transcription factor 27	GATGTCCTCGTCGTTGT	TATTTGCCCTGCTCCTCT
PGSC0003DMG400009968	25 kDa protein dehydrin	CAGCGTAACGTCCGTGT	GATGGCTTGTTCTGTTCG
PGSC0003DMG400006214	Caffeoyl-CoA O-methyltransferase 5	GGCAATGGACATTAACCG	TCAAGAAGAGGCAAAGCAG
PGSC0003DMG400007624	Early responsive to dehydration 15	TGGAGGAAGGTCATCAACAC	TTCGGGAAGCAAGTCAGC
